# Hydrophobicity is a key determinant in the activity of arginine-rich cell penetrating peptides

**DOI:** 10.1038/s41598-022-20425-y

**Published:** 2022-09-25

**Authors:** Jason Allen, Jean-Philippe Pellois

**Affiliations:** 1 Department of Biochemistry and Biophysics, Texas A&M University, Biochemistry and Biophysics Bldg., Room 430, 300 Olsen Blvd, College Station, TX 77843-2128 USA; 2 Department of Chemistry, Texas A&M University, College Station, TX 77843 USA

**Keywords:** Lipids, Peptides, Protein transport, Protein translocation

## Abstract

To deliver useful biological payloads into the cytosolic space of cells, cell-penetrating peptides have to cross biological membranes. The molecular features that control or enhance this activity remain unclear. Herein, a dimeric template of the arginine-rich HIV TAT CPP was used to establish the effect of incorporating groups and residues of various chemical structures and properties. A positive correlation is established between the relative hydrophobicity of these additional moieties and the ability of the CPP conjugates to deliver a peptidic probe into live cells. CPP conjugates with low hydrophobicity lead to no detectable delivery activity, while CPPs containing groups of increasing hydrophobicity achieve intracellular delivery at low micromolar concentrations. Notably, the chemical structures of the hydrophobic groups do not appear to play a role in overall cell penetration activity. The cell penetration activity detected is consistent with endosomal escape. Leakage assays with lipid bilayer of endosomal membrane composition also establish a positive correlation between hydrophobicity and membrane permeation. Overall, these results indicate that the presence of a relatively hydrophobic moiety, regardless of structure, is required in a CPP structure to enhance its cell penetration. It also indicates that simple modifications, including fluorophores used for cell imaging or small payloads, modulate the activity of CPPs and that a given CPP-conjugate may be unique in its membrane permeation properties.

## Introduction

Cell-penetrating peptides (CPPs) can achieve the delivery of useful molecular payloads inside mammalian cells, and hence potentially enable various cell biology, biotechnology, or therapeutic applications. When compared to other delivery vectors such as viruses or lipid nanoparticles, CPPs present several advantages, including ease of production, simplicity in formulation, and compatibility with a wide variety of payloads (protein, DNA, RNA, nanoparticles, etc.). Current drawbacks however involve relatively low cytosolic delivery efficiencies and a general lack of understanding of the factors that control or limit cell penetration^1–4^. Overall, structure–activity relationships that would help establish how CPP composition and structure influence membrane translocation and cell entry are still needed.

The HIV TAT peptide is a prototypical arginine-rich CPP that has been widely used as a delivery vector^5,6^. For instance, TAT has been conjugated to an enzyme such as Cre recombinase, and, upon external administration of TAT-Cre to cells for relatively long periods of time (24 h ≤), successful intracellular DNA recombination by TAT-Cre has been observed in a population of cells that may vary from 20 to 70%^7–9^. Notably, microscopy observations indicate that while a portion of TAT-Cre has certainly reached the nucleus, the vast majority of the protein is trapped inside endosomes (this is often observed as fluorescent puncta present in cells). These observations have been made with numerous payloads^10–12^. To date, despite cytosolic access being required for the biologic activity of most delivered payloads, the quantity of molecules of TAT/payload that reach the cytosol of cells, in addition to how cytosolic entry is achieved, remains relatively unclear. Several models for cell entry have been proposed and they typically include mechanisms by which CPP-payloads directly cross the plasma membrane of cells (direct plasma membrane translocation), and mechanisms by which CPP-payload are first endocytosed by cells, accumulate inside endosomes, and subsequently cross the membrane of endosomes (endosomal escape)^13–17^. Notably, these mechanisms are not necessarily incompatible with one another, and multiple routes of entry may co-exist, with dependence on CPP concentration, cell type, membrane oxidation, and payload size, among other factors.

Several strategies have been pursued to improve the delivery properties of CPPs like TAT. They include addition of endosomolytic peptide sequences, addition of hydrophobic residues and cyclization^18^. We and others have shown that reagents which combine multiple copies of the TAT peptide, or TAT-like analogs, are also more efficient at entering cells than their monomeric TAT counterpart. For instance, a simple dimer of TAT, dfTAT, yields high levels of endosomal escape^19^. In particular, protein payloads co-incubated with dfTAT reach the cytosol of cells by escaping late endosomes specifically, and the amount of protein that remains trapped inside endosomes can be less than 10% of what has entered the cytosol. In turn, this leads to intracellular payload activities that are many folds higher than what is achieved with TAT delivery. In addition, cell penetration and payload delivery is also a relatively fast process that takes place in less than an hour (i.e. a time-frame required for dfTAT to reach late endosomes via endosomal maturation)^20^. dfTAT contains two copies of TAT (RKKRRQRRR), linked by a cystine disulfide bridge and labeled with two tetramethylrhodamine fluorophores conjugated to the side chain of lysine residues ((CK(TMR)RKKRRQRRR)2). The arginine residues present in dfTAT play a major role in the endosomal activity of this peptide construct. A dfTAT analog that contains 10 arginine residues (2XR5 dimer) is relatively inefficient, while a construct that contains 12 arginine residues (2XR6 dimer) recapitulates the activity of dfTAT^21^. The spatial arrangement of R residues in the dfTAT construct, in addition to their number, may also play a role in peptide trafficking and cell entry. This is based on experiments establishing that peptides that contain a similar number of arginine residues assembled in a linear manner (TMR-R11 and TMR-R13) appear to favor direct plasma translocation over endosomal escape as a mechanism of cell entry^17^. Notably, all the constructs used in the structure–activity relationship studies described were labeled with TMR to facilitate visualization of the peptide localization in cells by fluorescence microscopy. However, this fluorophore was recently shown to participate in the cell penetration of dfTAT^22,23^. In particular, analogs lacking the fluorophore show a sharp decline in their ability to deliver payloads into cells. Herein, our goal was to establish structure–activity relationships in regard to the role played by the TMR moieties. Given that the addition of hydrophobic residues has been shown to impact the activity of monomeric TAT, and given that TMR is relatively hydrophobic, the hypothesis that hydrophobicity modulates the cell delivery activity of dfTAT-like analogs was tested^24,25^.

## Results

### Peptide design

**Figure 1:**
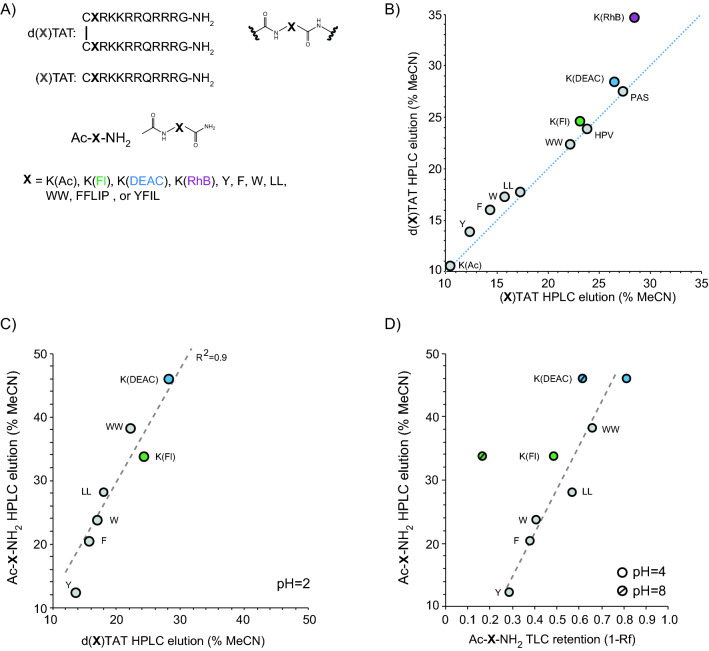
d(X)TAT peptides and their corresponding hydrophobicity. (**A**) General structure of the d(X)TAT constructs and of the Ac-X-NH2 analogs. X consists of the lysine residue labeled with on its side chain with an acetyl group (Ac), carboxyfluorescein (Fl), 7-diethylaminocoumarin-3-carboxylic acid, or rhodamine B. Alternatively, X consists of the residues tyrosine (Y), phenylalanine (F), tryptophan (W), or the peptide sequences LL, WW, FFLIP, or YFIL (where L is leucine, P proline, and I isoleucine). (**B**) Relative hydrophobicity of (X)TAT (monomeric peptide) and d(X)TAT (dimeric peptide formed by disulfide bond formation) based on C18 reverse phase HPLC. The percentage of acetonitrile at which the respective peptide elutes from the column is used as a measure of hydrophobicity. (**C**) Correlation between the hydrophobicity of d(X)TAT and corresponding Ac-X-NH2 analogs, as determined by C18 reverse phase HPLC. (**D**) Correlation between the hydrophobicity of Ac-X-NH2 determined by reverse phase HPLC (pH 2), or by reverse phase TLC (1-Rf displayed) at either pH 4 or pH 8. The TLC retention of Ac-X-NH2 with X = Y, F, W, LL, WW is pH independent and only results for pH 4 are shown.

In order to test how TMR contributes to the cell penetration activity of the peptide, we synthesized a small library of d(X)TAT analogs where X represent moieties that have some, but not all, of TMR properties (Fig. 1A). These moieties include a lysine (K) residue conjugated to an acetyl group (Ac), carboxyfluorescein (Fl), rhodamine B (RhB), or 7-diethylaminocoumarin-3-carboxylic acid (DEAC). The fluorophore TMR, Fl, and RhB share a similar xanthene core, but differ in charge and conjugation chemistry. The incorporation of RhB into a dfTAT analog has been reported to generate a non-fluorescent delivery tool (RhB loses fluorescence upon conjugation)^23^. DEAC is a coumarin-based fluorophore. X was also used to introduce a single canonical amino acid residue (F, Y, W), two residues (LL, WW), or short peptide sequences (YFIL or FFLIP). Like TMR, the residues F, Y, and W contain hydrophobic aromatic rings. The residue L is on the other hand aliphatic. The sequences YFIL and FFLIP have been reported as membrane destabilizing. YFIL is present in the human papillomavirus (HPV33) protein L2, a capsid protein involved in endosomal escape^26^. Notably, as in the d(X)TAT construct, YFIL is adjacent to an arginine-rich sequence in L2. Likewise, FFLIP, referred to a penetration accelerating sequence (Pas), has been reported to enhance the delivery of arginine-peptides^25^. All peptides were assembled by solid-phase peptide synthesis and assembled as CXRKKRRQRRRG. The monomeric peptides were then dimerized by disulfide bond formation (Figure S1–S10).

In order to assess the hydrophobicity contributed by each X group, the peptides were first analyzed by reverse phase HPLC using a C18 column, using gradients of buffer A (water/TFA 99.9:0.1) and buffer B (acetonitrile/water/TFA 89.9:10:0.1 v:v:v) (gradient of 0–40% B over 20 min). Based on this analysis, K(Ac) is the least hydrophobic X group, while K(RhB) is the most hydrophobic (Fig. 1B). All d(X)TAT peptides eluted over a range of 10–35% acetonitrile. The dimeric constructs were generally more hydrophobic than their monomeric counterparts, the hydrophobicity of (K(RhB))TAT being the most impacted by dimerization. One caveat of this approach is that the buffer system used for peptide analysis is pH 2 (due to the presence of TFA, required to protonate residue side chains). However, pH 2 is not representative of the pH that CPPs may be exposed to when in contact with cells, either pH 7.2 outside the plasma membrane, or pH between 6.5 and 4.5 while inside acidic endosomes. In order to establish the relative hydrophobicities of d(X)TAT peptides at these pHs, we sought to use other techniques, including thin layer chromatography using C18, aluminum oxide or silica stationary phases. Unfortunately, the arginine-rich peptides could not be separated on these systems. To solve this problem, each individual X group was synthesized, using acetylated and amidated N and C-terminal positions to mimic the peptide backbone found in the d(X)TAT constructs (Fig. 1A, Figure S11). Each Ac-X-NH2 group was analyzed by HPLC and by RP18 TLC, using 40% acetonitrile and 60% PBS as mobile phase. The HPLC elutions of Ac-X-NH2 correlated with the HPLC elutions of their respective d(X)TAT counterparts (Fig. 1C). Similarly, the relative hydrophobicities of Ac-X-NH2 determined by TLC (measured as 1-Rf) at pH 4 or 8 were in good agreement with the results obtained by HPLC (Fig. 1D). The TLC retention of Ac-X-NH2 was independent of pH for X = Y,F,W, LL and WW. Two exceptions were Ac-Fluo-NH2 and Ac-K(DEAC)-NH2, both displaying higher hydrophobicity at pH 4 than at pH 8. For fluorescein, this is consistent with the well-characterized pH-dependent absorption and fluorescence emission of the fluorophore, equilibria existing between dianionic, monoanionic, neutral and cationic forms of the molecule (with respective pKa of 6.4, 4.3 and 2.1)^27^. In the case of DEAC, the protonation state of the fluorophore has, to our knowledge, not been characterized.

### Moieties at position X impact the delivery of a peptide probe

While some d(X)TAT peptides are fluorescent, several d(X)TAT constructs are non-fluorescent and cannot be tracked inside cells directly. In order to compare all d(X)TAT, we chose to use the delivery of a fluorescent probe as a proxy for detecting cell entry. This fluorescent probe is the polylysine peptide k5 (lower case k indicating that the residues are D amino acids) labeled with TMR. TMR-k5 is readily internalized within endosomes by cells and lacks endosomal escape activity on its own (Figure S12). However, if released from endosomes by membrane-destabilizing agents, TMR-k5 can diffuse into the cytosol and stain nuclei and nucleoli^28^. Alternatively, transient permeabilization of the plasma membrane can also potentially allow TMR-k5 to enter cells and yield nuclear/nucleolar staining. Herein, we use this signal to establish intracellular access. We also chose to use TMR-k5 because this cationic polylysine peptide is unlikely to interact with cationic polyarginine CPPs like d(X)TAT because of electrostatic repulsion.

**Figure 2:**
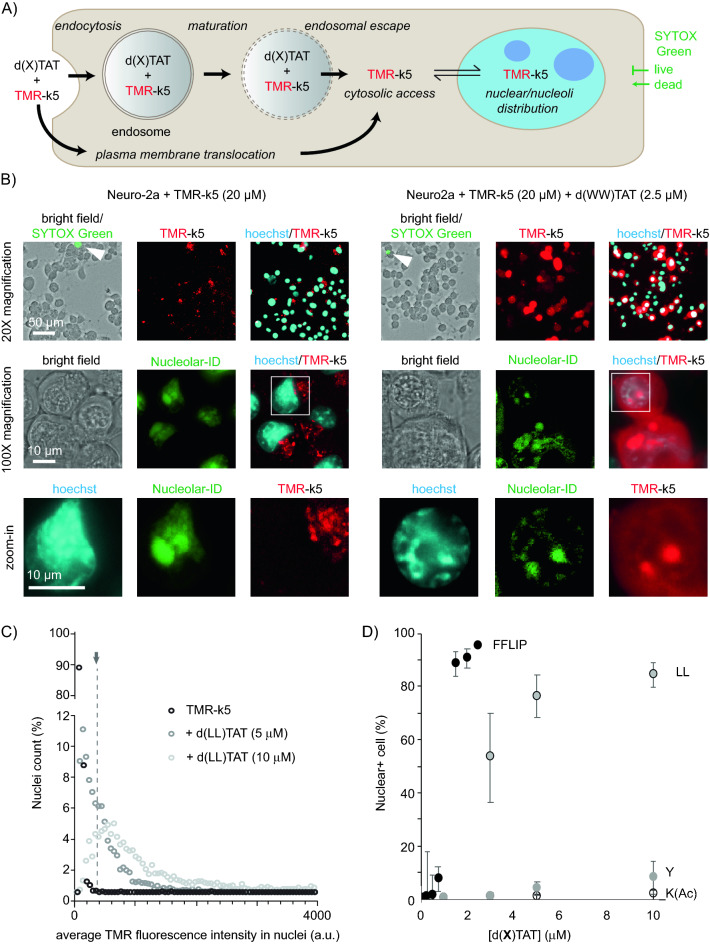
Assay used to assess the cellular delivery activity of d(X)TAT peptides. (**A**) Scheme highlighting two potential routes by which d(X)TAT can promote the cell entry of the probe TMR-k5. Cell entry is measured by monitoring the nuclear accumulation of TMR-k5 while the viability of cells is determined by exclusion of SYTOX Green. (**B**) Live cell microscopy images at ×20 and ×100 magnification of Neuro-2a cells incubated with TMR-k5 (20 μM) in the presence or absence of d(WW)TAT (2.5 μM). Bright field and SYTOX Green are overlaid in ×100 images, presence of dead cells being highlighted with white arrows. The nucleolar accumulation of TMR-k5 is highlighted in ×20 images by colocalization with the DNA stain Hoechst and the nucleolar stain Nucleolar-ID green. Zoom-in inset of the nuclear region are shown. (**C**) Representative results obtained from processing of fluorescence microscopy images. For each experiment, nuclei are identified from the Hoechst channel and the corresponding TMR intensity detected in these regions is measured. Cells incubated with TMR-k5 alone are used to establish a nuclear fluorescence background threshold (indicated by grey arrow). Cells incubated with TMR-k5 and d(X)TAT (here X = LL) containing nuclear red fluorescence above this determined threshold are considered positive for delivery (if also excluding SYTOX green) and are counted. (**D**) Quantified delivery efficiency of TMR-k5 in MDA-MB-231 cells when incubated with d(X)TAT peptides for 1 h at different concentrations. The plots provided are the averages and corresponding standard deviations of biological triplicates processed as described in (**C**). Results are presented for four representative d(X)TAT peptides where X is K(Ac), Y, LL, and FFLIP.

To test the relative delivery efficiency of the d(X)TAT peptides, the cell lines MDA-MB-231 (epithelial-like, human breast cancer, spindle-shaped) and Neuro-2a (mouse neuroblastoma with neuronal and amoeboid stem cell morphology) were chosen. These cell lines are routinely used in cancer research, neuroscience, and toxicology. In the context of this study, we rationalized that the distinct differences in origin and characteristics between MDA-MB-231 and Neuro-2a properties may be useful to assess the generality of the results obtained. MDA-MB-231 or Neuro-2a cells were incubated with d(X)TAT peptides at various concentrations and TMR-k5 (20 μM) for 1 h. Cells were then washed, incubated with the DNA stain Hoechst and the live/dead marker SYTOX green (or SYTOX blue), and imaged by fluorescence microscopy. The localization of TMR-k5 in individual nuclei was then assessed by measuring the overlap between TMR and Hoechst signals (Fig. 2, Figure S13). This assay therefore does not measure the number of TMR-k5 peptides delivered per cell, but instead the number of cells where TMR-k5 accesses nuclei/nucleoli above a detection threshold dictated by the concentration of peptide used and the microscope set up used (Figure S13). This is because, in our experience, dfTAT and dfTAT analogs display a threshold behavior where, below a certain endosomal concentration, endosomal leakage is not achieved. In contrast above this threshold most late endosomes are permeabilized. Hence, whether a payload is delivered in a given cell tends to be binary: it either escapes from endosomes or not, as opposed to the amount of payload leaking out from endosomes steadily increasing. Our rationale was therefore to titrate cells with different concentrations of d(X)TAT agents, monitor how many cells display cytosolic/nuclear delivery of TMR-k5, and compare the different d(X)TAT peptides.

**Figure 3:**
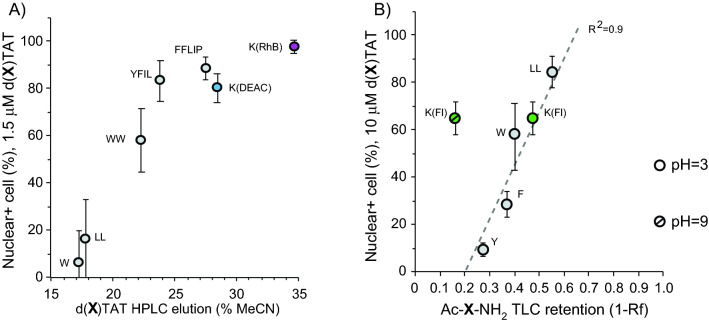
Correlation between CPP hydrophobicity and cell delivery. (**A**) Percentage of cells displaying TMR-k5 fluorescence in their nuclei as a function of the hydrophobicity of d(X)TAT. MDA-MB-231 cells were incubated with d(X)TAT (1.5 μM) and TMR-k5 (20 μM) for 1 h. The delivery data are the average and corresponding standard deviations obtained from biological triplicates. (**B**) Correlation between the delivery activity of d(X)TAT peptides and the hydrophobicity of the corresponding Ac-X-NH2 analogs. MDA-MB-231 cells were incubated with d(X)TAT (10 μM) and TMR-k5 (20 μM) for 1 h.

The percentage of cells displaying TMR-k5 nuclear staining varies depending on the group X, and on the concentration of d(X)TAT peptide used (Fig. 2). Overall, three general categories of d(X)TAT peptides can be identified: peptides (X = K(Ac), Y, F, W) where little to no TMR-k5 delivery is detected, peptides (X = LL, K(Fl)) where delivery is achieved in a high percentage of cells when a relatively high d(X)TAT concentration is used (> 5 μM), and peptides (X = WW, YFIL, DEAC, FFLIP, RhB) where delivery is achieved in a high percentage of cells at relatively low d(X)TAT concentrations (< 3 μM) (Fig. 2D). The TMR-k5 delivery activity of the d(X)TAT analogs was correlated to their relative hydrophobicity, as measured by either HPLC or TLC of the corresponding X groups (Fig. 3). These correlations were established at a concentration of d(X)TAT of 1.5 μM, a concentration at which “high activity” analogs display a range of activity, and at 10 M, a concentration at which the “low activity” analogs are distinguishable. A positive correlation is observed between d(X)TAT-mediated and the hydrophobicity of the X group at these concentrations (Fig. 3). A noticeable outlier is K(Fluo) when using the hydrophobicity of the corresponding Ac-X-NH2 group established at pH 9. However, the more hydrophobic form of the fluorophore detected at pH 3 follows the trend established with other X groups (Fig. 3B). The structure–activity relationships between the various d(X)TAT analogs and TMR-k5 delivery were similar between MDA-MB-231 and Neuro-2a, delivery efficiency being generally lower in the latter (Figure S14).

### The hydrophobicity of X impacts lipid bilayer leakage in vitro

**Figure 4:**
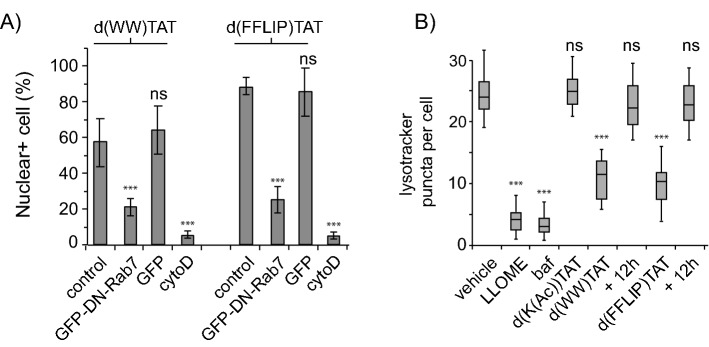
(**A**) Quantification of TMR-k5 nuclear delivery by d(X)TAT (X = WW or FFLIP) in Neuro-2a cells transfected with GFP-DN-Rab7 or GFP. TMR-k5 nuclear delivery is quantified as in Fig. 2. The control condition represents non-transfected cells. The data represents the average of biological triplicates and the corresponding standard deviations. The label ns corresponds to p > 0.05, *** corresponds to p < 0.001, p values being determined using a two-tailed *t* test. (**B**) Quantification of lysotracker-green stained puncta before and after incubation with d(X)TAT (X = K(Ac), WW, or FFLIP) at 10, 1.5, and 1.5 μM, respectively) for 1 h. After washing, cells are stained with LysoTracker Green DND-26 (500 nM) for 5 min. The number of green puncta in cells imaged at ×100 magnification is then counted in 100 cells per experiment. The data are represented as box-and-whisker plots, obtained from biological triplicates. Controls include LLOME, a compound that causes endosomal membrane damage, and bafilomycin, an inhibitor of the vacuolar H^+^ ATPase. For cells incubated with d(X)TAT peptides, the number of lysotracker puncta was established 1 h or 12 h after incubation to assess cell recovery.

Given that hydrophobicity of the d(X)TAT peptides appears to contribute to their membrane permeation activities, we next sought to establish whether this relationship could be recapitulated with membrane models, i.e. lipid bilayers. To establish which membrane system to test, we first aimed to test whether d(X)TAT peptides generally recapitulate the mechanism of cell entry previously determined with dfTAT. For this purpose, the delivery of TMR-k5 was performed in cells transfected with dominant-negative (DN) rab7(T22N), a protein that disrupts trafficking from early endosomes to late endosomes^29,30^. For these experiments, d(WW)TAT and d(FFLIP)TAT were chosen as two “high activity/hydrophobicity” d(X)TAT representatives. The nuclear staining of TMR-k5 delivered with the two d(X)TAT peptides was markedly reduced in cell transfected with EGFP-DN-rab7 but not in cells transfected with EGFP (Fig. 4A). Delivery was nonetheless observed in 20% of cells (these cells express the EGFP-DN-rab7 construct based on the EGFP fluorescence signal), indicating that endosomal maturation was not fully inhibited in these cells or alternatively, that cell entry does not solely involve late endosomes in this subpopulation. Delivery of TMR-k5 was also significantly reduced in the presence of cytochalasin D (cytoD, 20 μM), an inhibitor of actin dynamics and macropinocytic uptake (Fig. 4A)^31,32^. In this case, approximately 5% of cells remained positive for TMR-k5 nuclear staining. As with DN-rab7 experiments, this may suggest either incomplete inhibition of endocytosis by cytoD (cytoD does not fully inhibit several receptor-mediated uptake pathways) or endocytosis-independent cell entry by d(X)TAT/TMR-k5 in a relatively small population of cells^28^. To further probe the cell penetration mechanism, the number of endosomal organelles stained by Lysotracker in cells before and after delivery was quantified. The rationale behind this assay is that lysotracker accumulates in acidified organelles, typically late endosomes and lysosomes, that endosomal membrane leakage causes an increase of endosomal pH (presumably via leakage of luminal H^+^ into the cytosol), and that, consequently, endosomes rendered potentially leaky by d(X)TAT peptides will not be stained by lysotracker^28^. Consistent with these ideas, l-Leucine-l-Leucine methyl ester (LLOME), an endosomolytic agent, and bafilomycin, an inhibitor of the vacuolar H^+^-ATPase, both reduced the number of lysotracker stained puncta in treated MDA-MB-231 cells (Fig. 4B)^33–35^. d(K(Ac))TAT, a peptide with no detectable delivery activity, did not affect lysotracker staining. In contrast, d(WW)TAT and d(FFLIP)TAT caused a reduction in lysotracker stained endosomes, as assessed immediately after 1 h incubation with peptides (the reduction is less pronounced than with LLOME or bafilomycin, consistent with the notion that d(X)TAT peptides may disrupt only a subset of low pH organelles, i.e. late endosomes and not lysosomes). Notably, the number of lysotracker-positive puncta per cell was restored to control level when assessed 12 h after incubation with d(WW)TAT and d(FFLIP)TAT, suggesting that cells recover from the endosomal disruption that occurs upon delivery. Overall, these data indicate that endosomal escape is contributing to the delivery of TMR-k5 into cells. However, they do not rule out the possibility that direct plasma membrane translocation may also be involved as a concurrent phenomenon.

**Figure 5:**
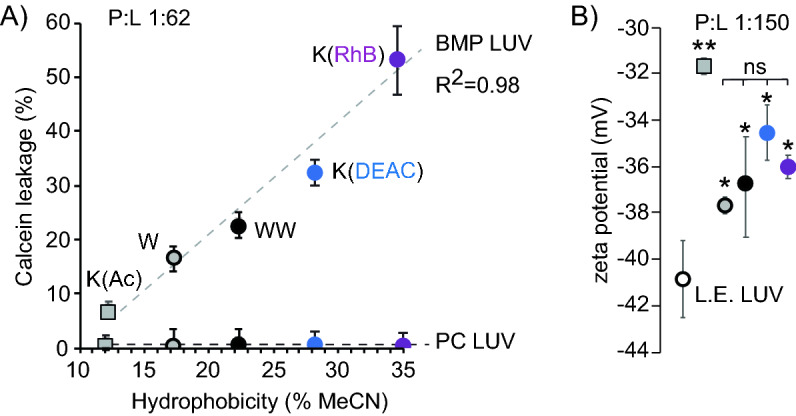
(**A**) Correlation between leakage assays and d(X)TAT hydrophobicity. Leakage assays were performed with calcein-loaded LUVs composed of zwitterionic lipids (PC LUV) or the anionic lipid BMP (BMP LUV). In all experiments, peptide to lipid ratio is 1:62. The data provided is the average and corresponding standard deviation of triplicate experiments. (**B**) Zeta potential of BMP LUVs in contact with d(X)TAT peptides, at a P:L of 1:150.

The interaction partners of CPPs that may mediate direct plasma membrane translocation remain undefined. It is therefore unclear which liposome system can be used to probe this activity. In contrast, the endosomal escape of TAT or dfTAT from late endosomes has been reported to involve the ionic lipid BMP^20,22^. This permeabilization is consistent with a leaky fusion process, where peptide-induced contact between liposomes is required for leakage. In order to assess whether similar processes are involved with peptides of the d(X)TAT series, LUVs were loaded with calcein to probe leakage. LUVs were prepared with either 65:15:20 PC:PE:Chol or 77:19:4 BMP:PC:PE, lipid compositions consistent with plasma membrane /early endosomes (PM/EE) bilayers or late endosome (LE) bilayers, respectively^36,37^. Peptides were added to liposomes suspensions at various peptide-to-lipid ratios, and the release of calcein was monitored by gel filtration. The d(X)TAT peptides did not cause release of calcein from PM/EE under the conditions tested but permeabilized LE LUVS (Fig. 5A). Notably, the leakage activity of individual d(X)TAT peptide positively correlated with the hydrophobicity of the X group (Fig. 5A), peptide:lipid ratio P:L is 1:62). In order to assess whether differences in leakage activity may relate to differences in d(X)TAT peptide association with BMP bilayers, zeta potential measurements were performed at a P:L of 1:150 (a condition that leads to no apparent leakage and no apparent change in liposome size distribution, as measured by dynamic light scattering; data not shown). Under these conditions, d(K(Ac))TAT had the largest effect on the apparent zeta potential of the negatively-charged BMP-rich LUVs (binding of the positively-charged peptide reduces the apparent negative zeta potential of the anionic BMP-rich liposomes). Other d(X)TAT peptides also reduced the zeta potential but were not statistically distinguishable from another in this respect. Notably, the effect of these peptides on zeta potential was less pronounced than with d(K(Ac)TAT. It is possible that d(K(Ac))TAT and other d(X)TAT peptides bind differently to the bilayers, hence changing the slipping plane and the resulting apparent zeta potential. Nonetheless, these results suggest that d(K(Ac))TAT does not fail to induce leakage simply because it is less capable of binding to the surface of LE LUVs than other d(X)TAT analogs. In turn, these data suggest that hydrophobic X groups may be more directly involved in interactions that promote leakage of the lipid bilayer.

## Discussion/conclusion

The delivery assay used in this report has advantages and disadvantages. One advantage is that it uses the same probe for all d(X)TAT peptides and, in principle, allows direct comparison. It also allows the monitoring of the delivery activity of d(X)TAT that are non-fluorescent and not directly visible by microscopy. We were not able to detect interactions between d(X)TAT peptides and TMR-k5 using various techniques (e.g. fluorescence anisotropy, fluorescence resonance energy transfer, data not shown). On one hand, it is not surprising as both d(X)TAT and TMR-k5 are polycationic and likely repulsing one another. It is therefore possible that TMR-k5 acts as a relatively inert fluid-phase probe, simply detecting leakage from a biological membrane transiently permeabilized by d(X)TAT. On the other hand, it is also possible that d(X)TAT and TMR-k5 interact when in contact with cells. Alternatively, they may bind to common anionic cellular partners, either simultaneously or in competition. It is also important to note that TMR-k5 contains TMR, the fluorophore used for labeling of the parent peptide dfTAT described in introduction. It is therefore important to consider that the absolute values in delivery efficiency presented herein are likely specific to the probe and assay used. The relative comparison between d(X)TAT and the correlation with the hydrophobicity of the X group are however likely to be applicable to the delivery of other payloads.

The enhancement of TAT’s cell penetration activity by addition of hydrophobic residues has been reported before^24,38^. The results presented herein for the dimeric d(X)TAT constructs are consistent with these previous reports. d(K(Ac))TAT, which is the lowest peptide on the hydrophobicity scale used in this report, does not have a delivery activity detectable in our assay. Previous results have indicated that arginine-rich sequences are necessary for cell penetration of dfTAT analogs and that a threshold of arginine residue content needs to be reached to obtain substantial endosomal escape^21^. The results presented herein suggest in turn that arginine residues alone are not sufficient to promote cell entry. Addition of various residues and fluorophores (X groups) can dramatically modulate the cell delivery and lipid bilayer activities of the peptides. The hydrophobicity of these residues, as determined by retention with C18 stationary phases, appears to be the main defining feature that controls cell penetration. Conversely, comparison between the X group suggests that no apparent structural feature influences cell penetration. For instance, the introduction of leucine, which has an aliphatic side chain, can recapitulate the activity obtained with residues or fluorophore containing bulky aromatic rings. The contribution of the hydrophobic K(DEAC) and K(RhB) residues is also comparable to the contribution provided by YFIL or FFLIP peptides, suggesting that peptide sequences and length are not determining factors. Instead, one may simply need to add multiple residues of relatively low hydrophobicity together to reach the hydrophobicity provided by the large organic fluorophores. Overall, these results highlight that, while the incorporation of a hydrophobic moiety in an arginine-rich CPP is important to enhance cell penetration, there is broad flexibility in what this hydrophobic moiety can be. Conversely, these results also indicate that groups conjugated to a CPP may have a significant impact on its cell penetration, whether it is a fluorophore used for imaging applications or whether it is a potential small molecule or peptide payload. In turn, this may contribute to a high variability in delivery outcomes when using different CPP-conjugates. In this context, an interesting result is that obtained with the CPP labeled with fluorescein, a fluorophore commonly used. The pH dependence of the fluorophore, with an equilibrium between a hydrophilic (high pH) and hydrophobic (low pH) forms of the fluorophore, can have a significant impact on the activity of the CPP. In particular, our results would predict that d(K(Fluo))TAT is likely less active outside cells (pH 7.2, hydrophilic/hydrophobic forms in a ~ 10:1 ratio based on Henderson-Hasselbach equation and pKa of ~ 6), and more hydrophobic and membrane active inside endosomes (pH 5, hydrophilic/hydrophobic forms in a ~ 1:10 ratio). Hence, this choice of fluorophore labeling may reduce the ability of the CPP to enter cells via direct plasma membrane translocation, while favoring endosomal escape. Notably, this low pH-triggered membrane activity is something that can be exploited to enhance the specificity of membrane active peptides towards endosomal membranes^9,39–43^.

The hydrophobicity of d(X)TAT peptides can in principle contribute to numerous aspects of the interactions between these CPPs and cellular macromolecules. On their journey to the cytosol of cells, hydrophobicity could in principle alter binding with various cell surface proteins, proteoglycans, and lipids, and influence both direct plasma membrane translocation and endocytic uptake. While it is possible that direct plasma membrane translocation is responsible for the delivery activity we detect in a small population of cells, cell-based assays that inhibit endocytic uptake or endosomal maturation indicate that endosomal escape is likely the predominant route of cell entry in the majority of cells. Inside endosomes, the hydrophobicity of d(X)TAT could influence proteolytic degradation by endosomal protease, interactions with endosomal membranes, and endosomal escape. Our in vitro assays with liposomes support the notion that this latter step may be facilitated by the incorporation of hydrophobic X groups in the d(X)TAT CPP structure. One can speculate that, while the cationic arginine residues present in the TAT peptide are likely primarily involved in interacting with lipid polar heads, a hydrophobic X group could potentially be involved in biophysical processes that involve the hydrophobic environment of a bilayer. It should be noted that hydrophobicity could in principle contribute several deleterious effects if increased to levels beyond those tested herein. In particular, it is likely that, above a currently undefined threshold, d(X)TAT analogs may be too disruptive to membranes and become broadly toxic. Additionally, very hydrophobic variants may aggregate and have poor solubility. Further studies will be required to more precisely dissect the contribution of hydrophobic groups in the step of CPP-mediated bilayer leakage, as well as in other processes contributing to cell penetration. It should also be important to identify optimal hydrophobicity ranges that consider the toxicity and handling properties of the peptides. Such studies may permit further refinement in the design of next generation CPPs.

## Materials and methods

### Peptide design, synthesis, purification and quantitation

All peptides were synthesized on a rink amide MBHA resin (Novabiochem, San Diego,CA) by solid phase peptide synthesis (SPPS) using previously described protocols^19^. The amino acids Fmoc-Lys-(Boc)-OH, Fmoc-Gly-OH, Fmoc-Arg(Pbf)-OH, and Fmoc-Gln-(Trt)-OH (Novabiochem) were used to construct the TAT sequence. Fmoc-Tyr(tBu)-OH, Fmoc-Phe-OH, Fmoc-Trp(Boc)-OH, Fmoc-Leu-OH, Fmoc-Ile-OH, Fmoc-Lys(Ac)-OH and Fmoc-Pro-OH were added where applicable for specific peptides. Fmoc-Lys(Mtt)-OH was used for peptides requiring fluorophore conjugation. For these peptides, Mtt was removed with 1.5% TFA while the peptide is attached to the resin. The fluorophores 7-diethylaminocoumarin-3-carboxylic acid (DEAC)(AnaSpec), carboxyfluorescein (Fl)(Sigma), or rhodamine B (RhB)(Sigma) were then coupled to the ɛ-amine of the deprotected lysine residue using standard coupling protocols. Boc-Cys(Trt)-OH was added to the N-terminus of all peptides. Peptides were then cleaved from the resin, purified by HPLC, and dimerization by disulfide bond formation, using previously reported protocols^19^. The TMR-k5 construct was synthesized using Fmoc-d-Lys(Boc)-OH with 5(6)-carboxytetramethylrhodamine (TMR) conjugated directly to the N terminus. Peptide substituents for TLC analysis were also synthesized in a similar manner, using acetic anhydride to cap the N terminus of resin-bound residues. All peptides were analyzed by reverse phase HPLC on an Agilent HP 1200 series instrument and an analytical Phenomenex Luna Omega Polar C18 column (5 μm, 4.6 mm × 250 mm). The flow rate was held at 2 mL/min, and detection was measured at 214 nm. Peptide purification was performed on a Thermo Fisher UltiMate 3000 HPLC on a Phenomenex Luna Omega Polar C18 column (5 μm, 21.2 mm × 250 mm). The flow rate was held at 20 mL/min, and pure peptide fractions were collected based on thresholds set for absorbance at either 214, 430, or 450 nm, dependent upon the absorbance of the peptide being purified. All runs used linear gradients of 0.1% aqueous TFA (solvent A) and 90% acetonitrile, 9.9% water, and 0.1% TFA (solvent B). Peptides were purified to at least 95% purity (based on presence of residual peaks detected at the same absorbance) and the correct identity of all peptides was confirmed by electrospray ionization on a Thermo Exactive Orbitrap (Figure S1–S11). The concentration of peptides was determined using quantitative amino acid analysis (Protein Chemistry Lab, TAMU) on the purified peptides.

### Hydrophobicity determination by HPLC

All rpHPLC retention times were analyzed using an Agilent HP 1200 series instrument on an analytical Phenomenex Luna Omega Polar C18 column (5 μm, 4.6 mm × 250 mm). The flow rate was 2 mL/min, and detection was at 214 nm. A binary solvent system was used comprising 99.9% H_2_O/0.1% TFA (solvent A) and 90% Acetonitrile/9.9% H_2_O/0.1% TFA (solvent B). Elutions were performed over a 20 min 0–40% solvent B gradient for all whole peptides (d(X)TAT), and a 20 min 0–100% solvent B gradient for acetylated hydrophobic substituents (Ac-X-NH_2_).

### Thin layer chromatography on acetylated Ac-(X)-TAT substituents

Hydrophobic substituents (10 µL) were developed on aluminum-backed F_254_ RP-18 TLC plates (EMD Millipore). Plates were cut into 6 cm × 6 cm squares and developed in 40% acetonitrile/60% 10 mM phosphate buffer first along one axis at pH 2.0, then along the second axis at pH 7.4. Peptide substituents that could not be easily identified under long-wave UV light were stained with iodine for 10–20 min. Retention factors (R_f_) were calculated based on the distance traveled by each compound between the starting spotted location and the final solvent front location.

### Delivery into live cells

MDA-MB-231 and Neuro2a cells obtained from ATCC (breast cancer-derived epithelial) were cultured in humidified atmosphere at 5% CO_2_ and 37 °C. Cells were seeded in plastic 48-well plates (Fisher) for quantitative analysis or 8-well Labtek II plates (Fisher) for qualitative imaging at 100× magnification. The cells were grown to 80–90% confluency in a 37 °C humidified atmosphere containing 5% CO_2_. Cells were washed three times with Leibovitz’s L15 media (L15) (Fisher). Cells were then incubated with peptide and 20 µM of the TMR-k5 pentalysine probe at 37 °C for 1 h. This was followed by washing the cells three times with L15 supplemented with heparin (Sigma-Aldrich) (1 mg/mL) and an extra L15 wash. Cells were treated with the cell impermeable nuclear stain SYTOX Blue (Thermo Fisher Scientific), SYTOX Green (Thermo Fisher Scientific), or DRAQ 7 (Invitrogen) to identify cells that had a compromised plasma membrane (i.e., dead cells). Additionally, the cell permeable dye Hoechst 33342 (Thermo Fisher Scientific o) was used for nuclear staining. Cells were imaged using an EVOS FL Auto 2 inverted microscope (Thermo Fisher Scientific). The microscope is equipped with a heating chamber maintained at 37 °C. Images were acquired using bright field imaging and five fluorescence filter sets: CFP (Ex = 436 ± 10 nm, Em = 480 ± 20 nm), RFP (Ex = 560 ± 20 nm, Em = 630 ± 35 nm), FITC (Ex = 488 ± 10 nm Em = 520 ± 20 nm), DAPI (Ex = 350 ± 50 nm, Em = 460 ± 25 nm), Cy5 (Ex = 350 ± 50 nm, Em = 460 ± 25 nm). For image viewing and processing individual images, the Celleste 4.0 software (Thermo Fisher Scientific) was used. Nuclear regions in 20× images where identified using Hoechst staining (the quantification process is described in Figure S13). The intensity of these objects in the TMR channel was then determined using the Celleste software. Cells stained by the SYTOX or DRAQ dyes were considered dead and not counted as positive for delivery. Cells displaying a nuclear TMR-k5 fluorescence above the fluorescence threshold determined for TMR-k5 alone were considered positive for delivery (if excluding SYTOX or DRAQ7 stain, and if visually showing stained nucleoli for confirmation of intracellular localization). The percentage of cells positive for TMR-k5 nuclear staining was then calculated, using Hoechst-stained cells to count the total number of cells present. Please note that this assay does not quantified the number of molecules that enter cells and reach the nucleus/nucleolus. Instead, this assay quantifies the number of cells in which the nuclear fluorescence of TMR-k5 is above a detection threshold. This threshold is dependent on the microscope, imaging conditions, and the concentration of TMR-k5 used during incubation (Figure S13). The reproducibility of all the experiments was assessed by performing experiments with independent batches of cell cultures on three different days (i.e., biological triplicates).

### Delivery inhibtion with dominant negative rab7 or CytoD

DN-rab7 (GFP-rab7 DN, Addgene plasmid # 12660) was a gift from Richard Pagano. A GFP gWiz expression vector was purchased from ALDEVRON. DNA plasmids were mixed with Lipofectamine 2000 reagent in opti-MEM medium and incubated at room temperature for 30 min. The DNA complex was added to previously seeded HeLa cells (85–90% confluent) on an eight-well dish, and cells were kept at 37 °C for 6 h. Cells were washed with fresh DMEM/10% FBS and cultured for an additional 24 h. Delivery experiments were then performed as described above. GFP fluorescence detected by fluorescence microscopy was used to identify cells expressing the GFP constructs (> 60% of total cells). For CytoD inhibition experiments, a stock of CytoD in DMSO (2 mM) was diluted in L15 culture media to 20 μM. Cells were incubated with CytoD (20 μM) for 30 min. Delivery experiments with d(X)TAT and TMR-k5 were then performed as described above, CytoD (20 μM) remaining present in media during the duration of the experiment (1 h).

### Staining of endosomes with LysoTracker

For the quantification of acidic vesicles, cells were treated with LysoTracker Green DND-26 (500 nM) for 5 min. Alternatively, cells were pretreated with L-Leu-L-Leu methyl ester (LLOME; Sigma-Aldrich) incubated at 1 mM for 10 min, or bafilomycin (Sigma-Aldrich) at 1 µM for 4 h, and then stained with LysoTracker. Likewise, cells were pretreated with d(X)TAT peptides (X = K(Ac), WW, or FFLIP at 10, 1.5, and 1.5 μM) for 1 h. Cells were then washed an stained with LysoTracker immediately or after 12 h incubation in DMEM. For all conditions, fluorescence microscopy images were acquired using the 100× objective on an Olympus IX-81 microscope. ImageJ was used to calculate the number of LysoTracker-stained puncta per cell, as well as the area of each individual puncta. This analysis was performed for 50 cells imaged for each condition and reproduced in biological triplicates.

### Liposome preparation

The lipids used in the experiments consisted of: 1,2-dioleoyl-s-glycero-3-phosphocholine (DOPC), 1,2-dioleoyl-sn-glycero-3-phosphoethanolamine (DOPE), sn-(3-oleoyl-2-hydroxy)-glycerol-1-phospho-sn-1′-(3′-oleoyl-2′-hydroxy-glycerol) (BMP), cholesterol (chol) (Avanti Polar Lipids). Liposomes were prepared by transferring the volume of lipids dissolved in chloroform (stock solutions of known concentrations) into scintillation vials. For liposomes mimicking the intraluminal late endosomes vesicles (L.E.) the molar ratios of lipids consisted of 77:19:4 BMP:PC:PE. For liposomes mimicking the plasma membrane or early endosomes, the lipid mixture was 65:15:20 PC:PE:Chol. The lipid film was prepared by removing the chloroform from the lipid mixture using a N_2_ (g) and then placing the vial in a desiccator overnight. To hydrate the lipids, buffer containing 100 mM NaCl, 10 mM NaH_2_PO_4_ pH7.4, with 70 mM calcein was added. The lipids were then mixed vigorously and swelled for 1 h at 42 °C under N_2_ to obtain multilamellar vesicles (MLVs). To obtain unilamellar vesicles (LUVs), the MLVS were extruded (20 passes) through a 100 nm pore size polycarbonate membrane (Whatman) using a Mini-Extruder (Avanti Polar Lipids). Dynamic light scattering was used to determine the average diameter size distribution of the liposomes using a Zeta Sizer Nano-s (Malvern). The liposomes encapsulated with calcein were purified by gel filtration using a Sephadex medium G-50 (GE Healthcare) column (2.5 × 17.5 cm) to separate the liposomes from free calcein. The eluate was collected in a 96 well plate and the plate was read using a Promega GloMax-Multi plate reader (Promega) at 450 nm and 750 nm corresponding to the wavelength of detection for calcein and liposome, respectively. Those wells containing purified calcein-encapsulated LUVs were consolidated and the final lipid concentration was determined based on the final volume relative to the starting volume with known concentration.

### Leakage assays

Leakage assays were carried out in 250 µL volumes at the specified ratios of peptide to lipid. Late endosomal assays were incubated in 100 mM NaCl, 10 mM NaH_2_PO_4_ at a pH of 5.5 to mirror conditions in the endocytic pathway, while plasma membrane leakage was measured at pH 7.4. Following a 1 h incubation at room temperature, reactions were centrifuged briefly at 1500×*g* (30 s), then for 2 min at 21,000×*g* to pellet flocculated lipids. To separate leaked calcein from remaining intact vesicles the supernatant was separated using a 10 × 1.5 cm column packed with Sephacryl 100-HR. The mobile phase was the same as the above buffer at pH 7.4 and was fed through the column at 3 mL/min using a Dionex UltiMate 3000 HPLC pump (Thermo Fisher Scientific). Percent leakage was calculated by integrating the calcein (488 nm) absorbance peak and comparing to the value obtained from LUVs solubilized with 0.2% Triton X-100.

### Zeta potential measurements

Peptides and LUVs of late endosomal composition were mixed together at 0.33 µM peptide to 50 µM lipid (ratio of 1:150 peptide:lipid) in PBS at pH 5.5. The mixtures were then added to a folded capillary zeta cell for measurement on a Zetasizer Nano-zs (Malvern). Instrument was set to hold at 25 °C and automatically determine the optimal voltage and attenuator positions for each measurement. Each sample was set to be measured three times by the instrument and the average of these measurements was used to determine the zeta potential for a single sample. This experiment was performed in triplicate for each condition.

## Supplementary Information

Supplementary Information.

## Data Availability

All data generated or analysed during this study are included in this published article and its supplementary information files.
