# Supplementary material for: Hydrophobicity is a key determinant in the activity of arginine-rich cell penetrating peptides
Source: Sci Rep. 2022 Sep 25;12:15981. doi: 10.1038/s41598-022-20425-y (PMC9510126; doi:10.1038/s41598-022-20425-y)

**Hydrophobicity is a key determinant in the activity of arginine-rich cell penetrating peptides**

Jason Allen^1^, & Jean-Philippe Pellois^1*^

From ^1^Department of Biochemistry and Biophysics, Texas A&M University, College Station, TX 77843, USA;

*To whom correspondence should be addressed

Address correspondence to: Jean-Philippe Pellois, Biochemistry and Biophysics Bldg., Room 430, 300 Olsen Blvd, College Station, TX, 77843-2128.

Fax: 979-862-4718, E-mail: [pellois@tamu.edu](mailto:pellois@tamu.edu)

**Figure S1-S10.** A) Structures, names and exact mass of d(X)TAT peptides used in this study The purity of each peptide is established by HLPC using detection of absorbance at 214 nm. Peptide masses are confirmed using electrospray ionization mass spectrometry. Impurities, when present, are highlighted by an asterisk.


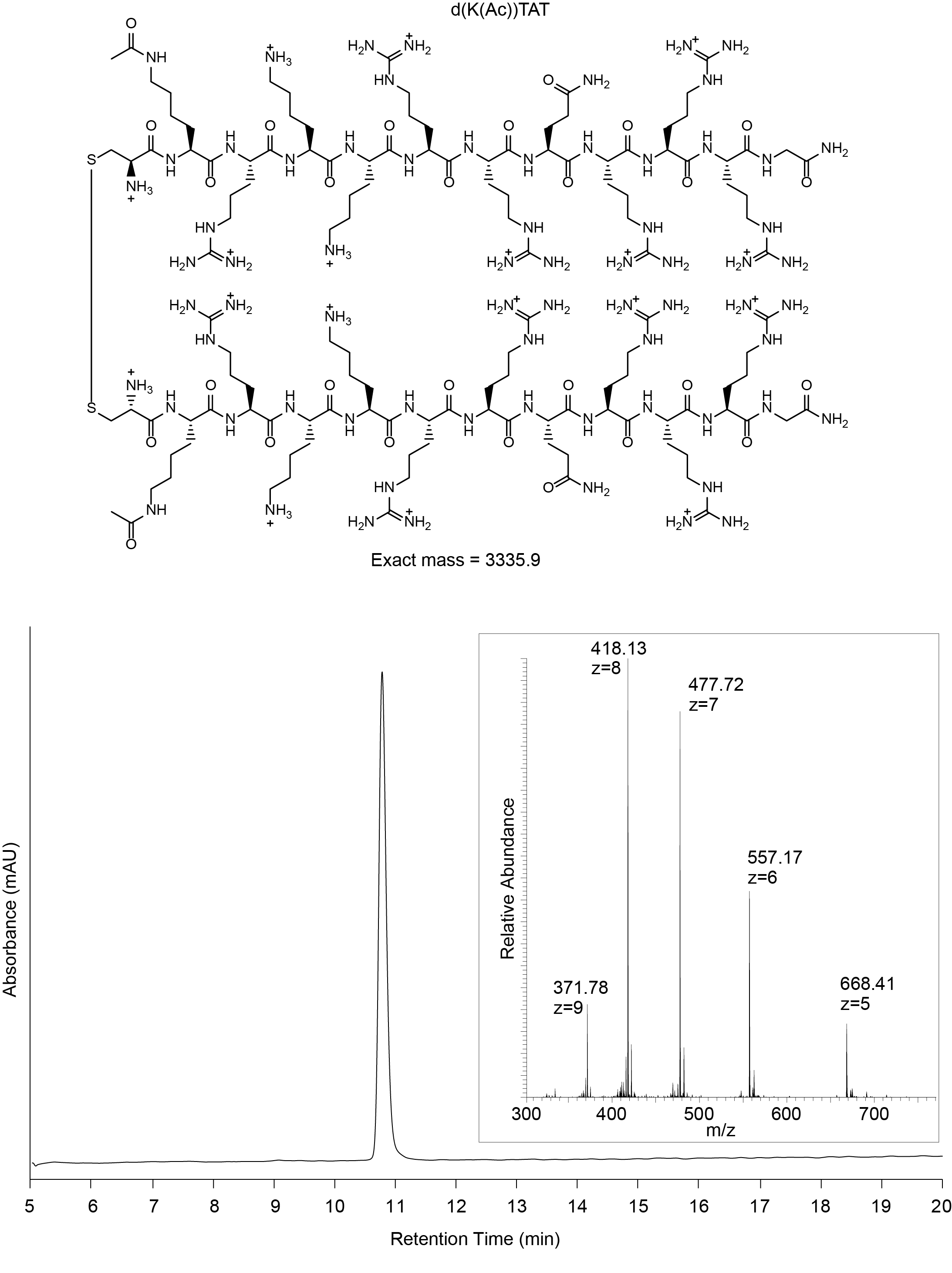


Figure S1.

Figure S2.


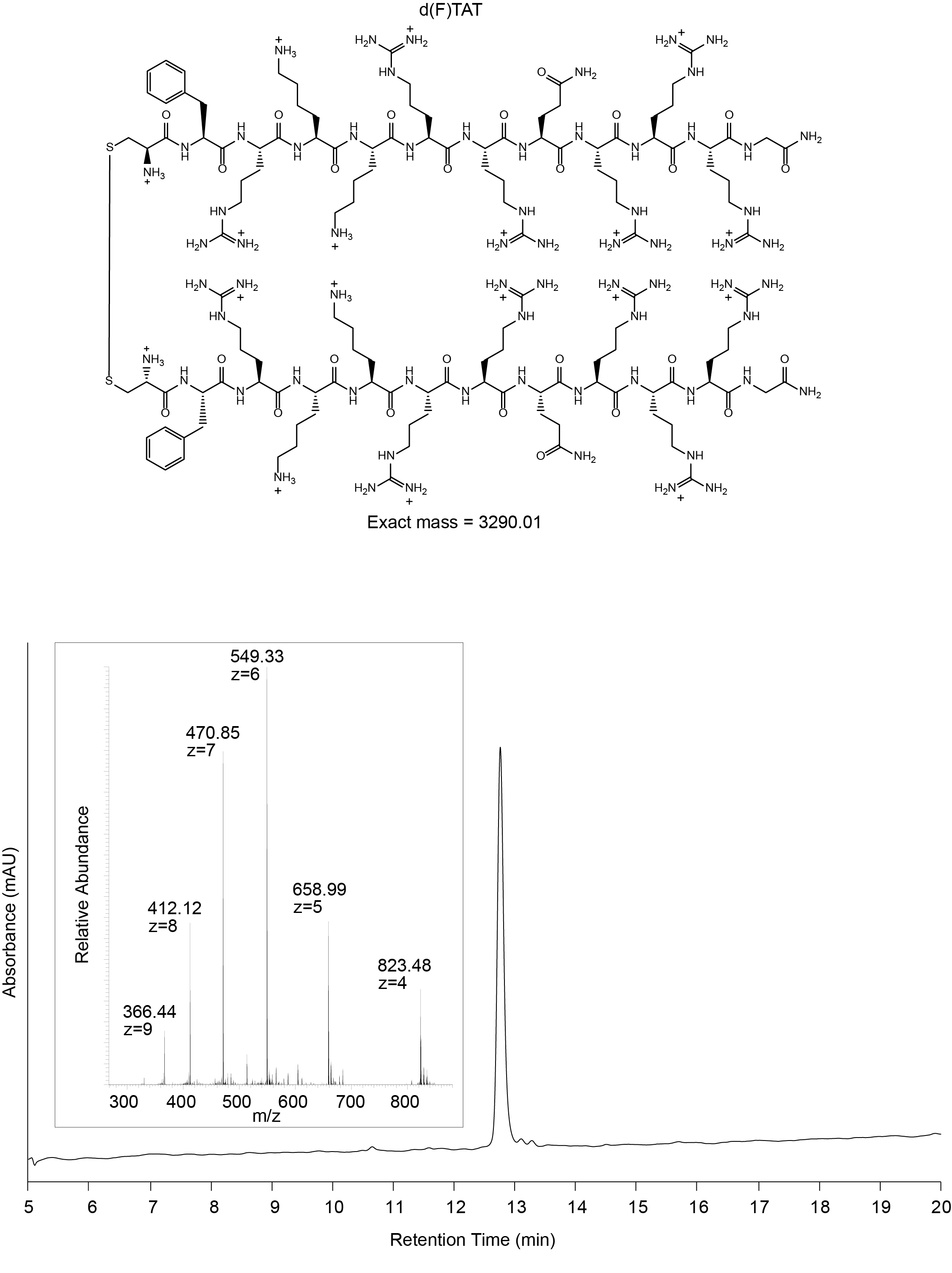


Figure S3.


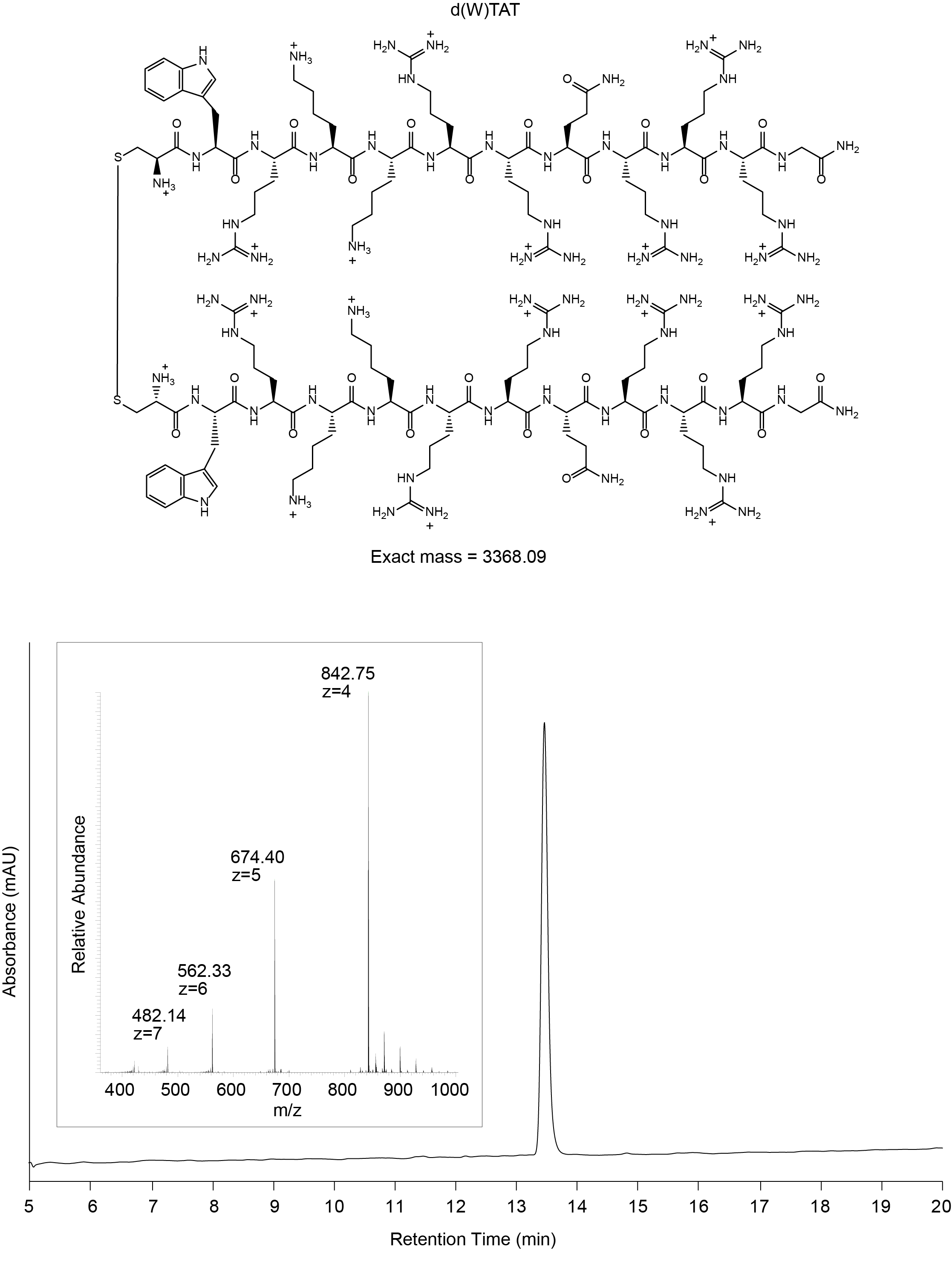


Figure S4.


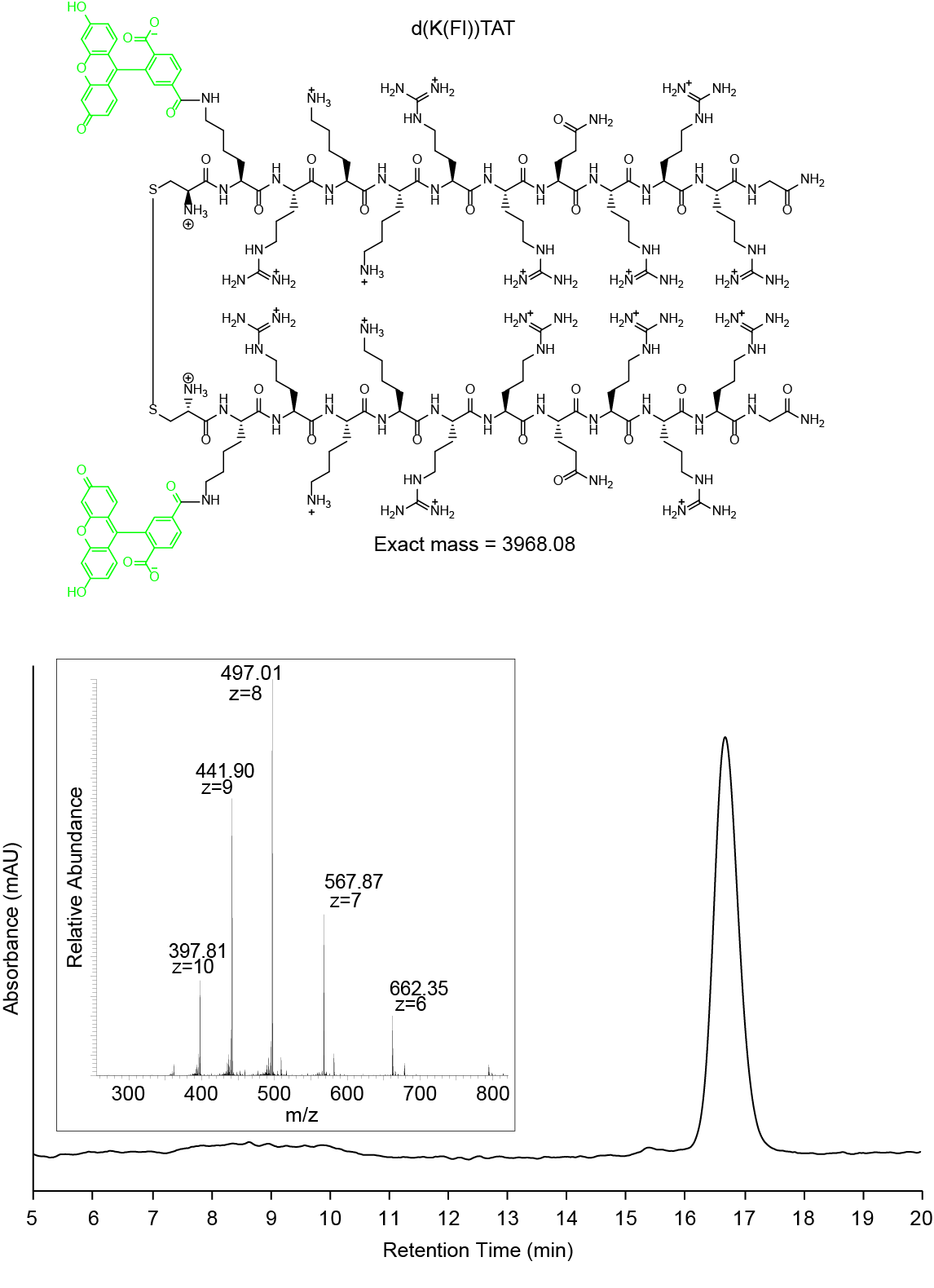


Figure S5.


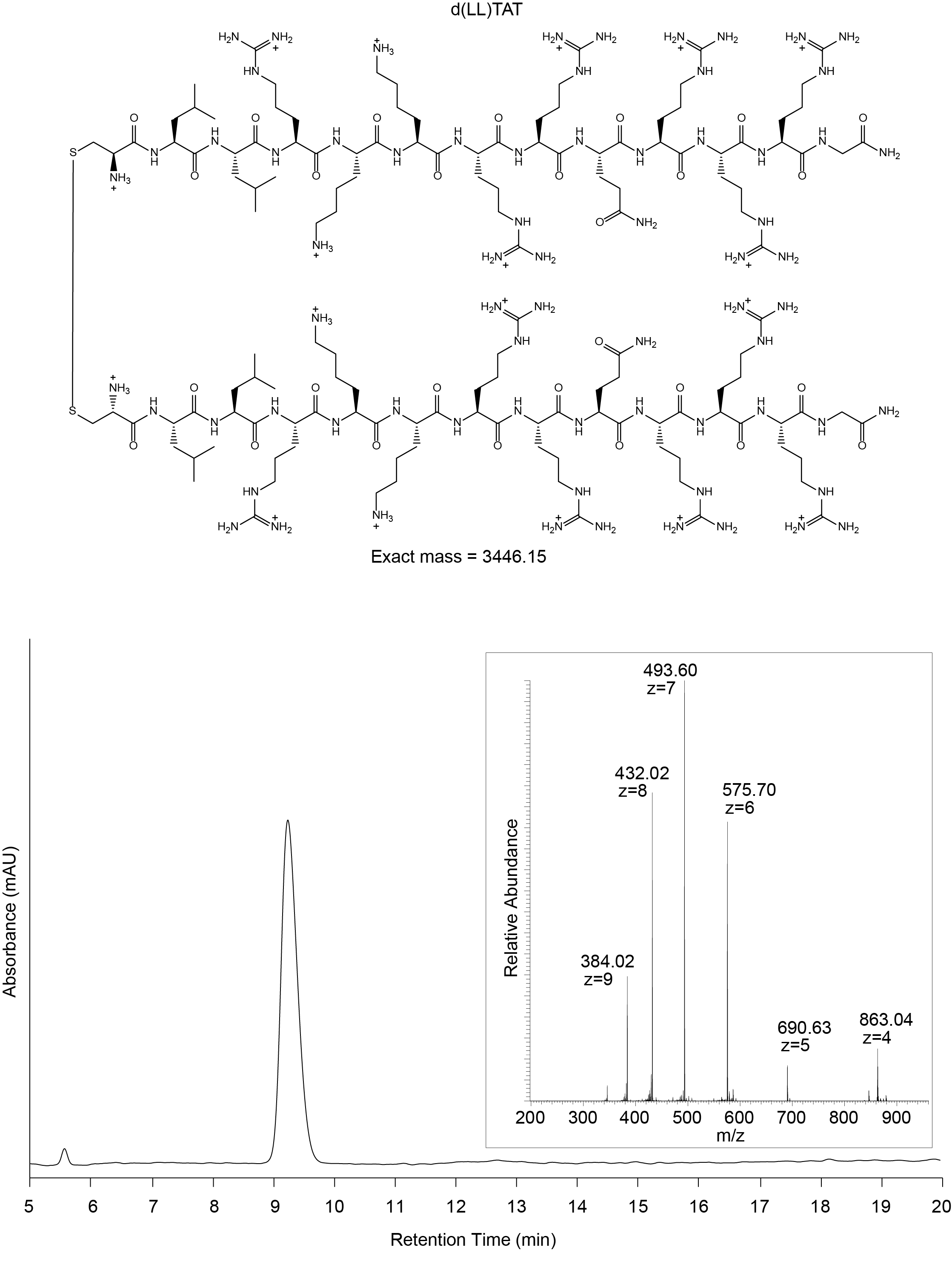


Figure S6.


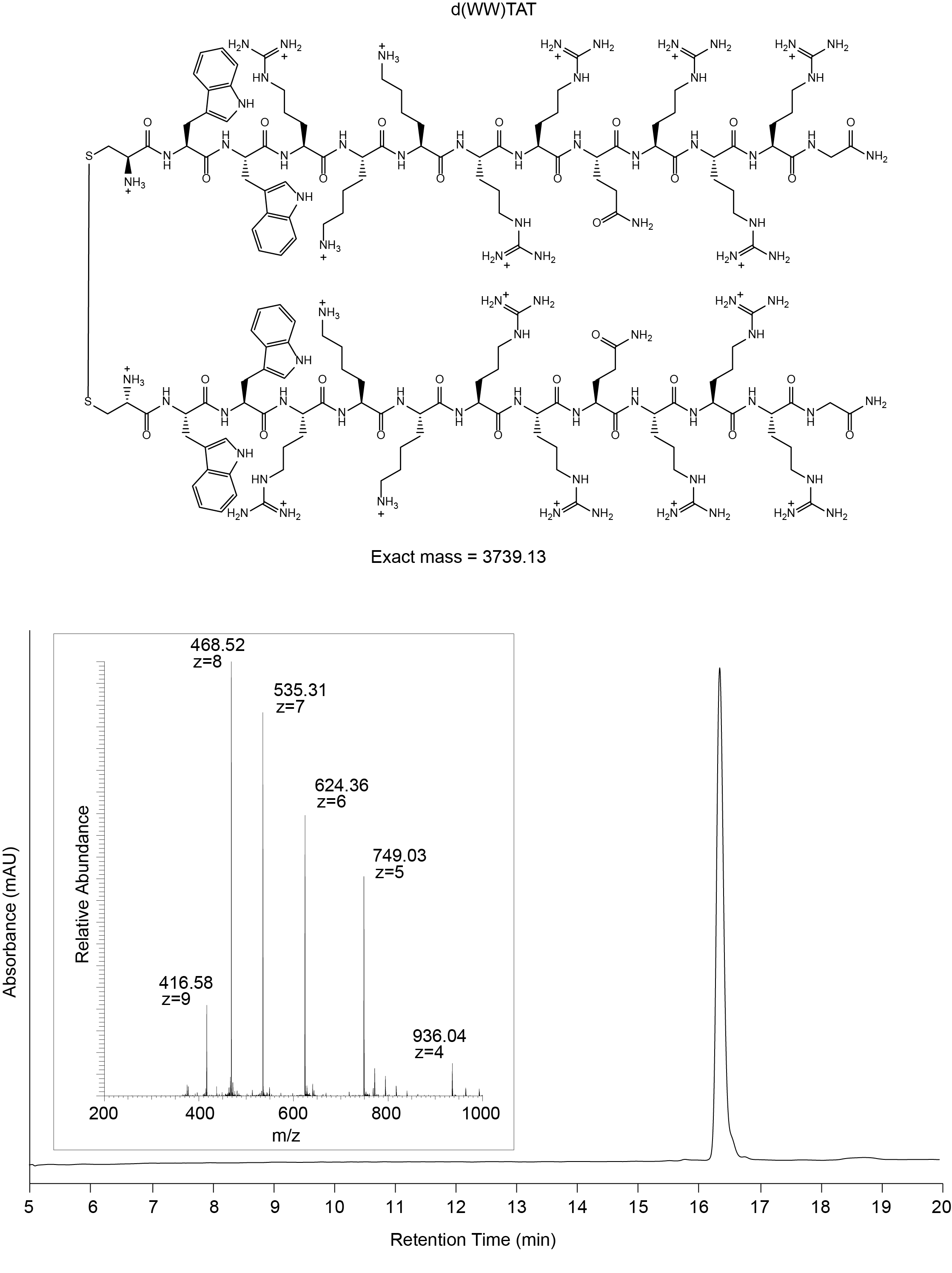


Figure S7.


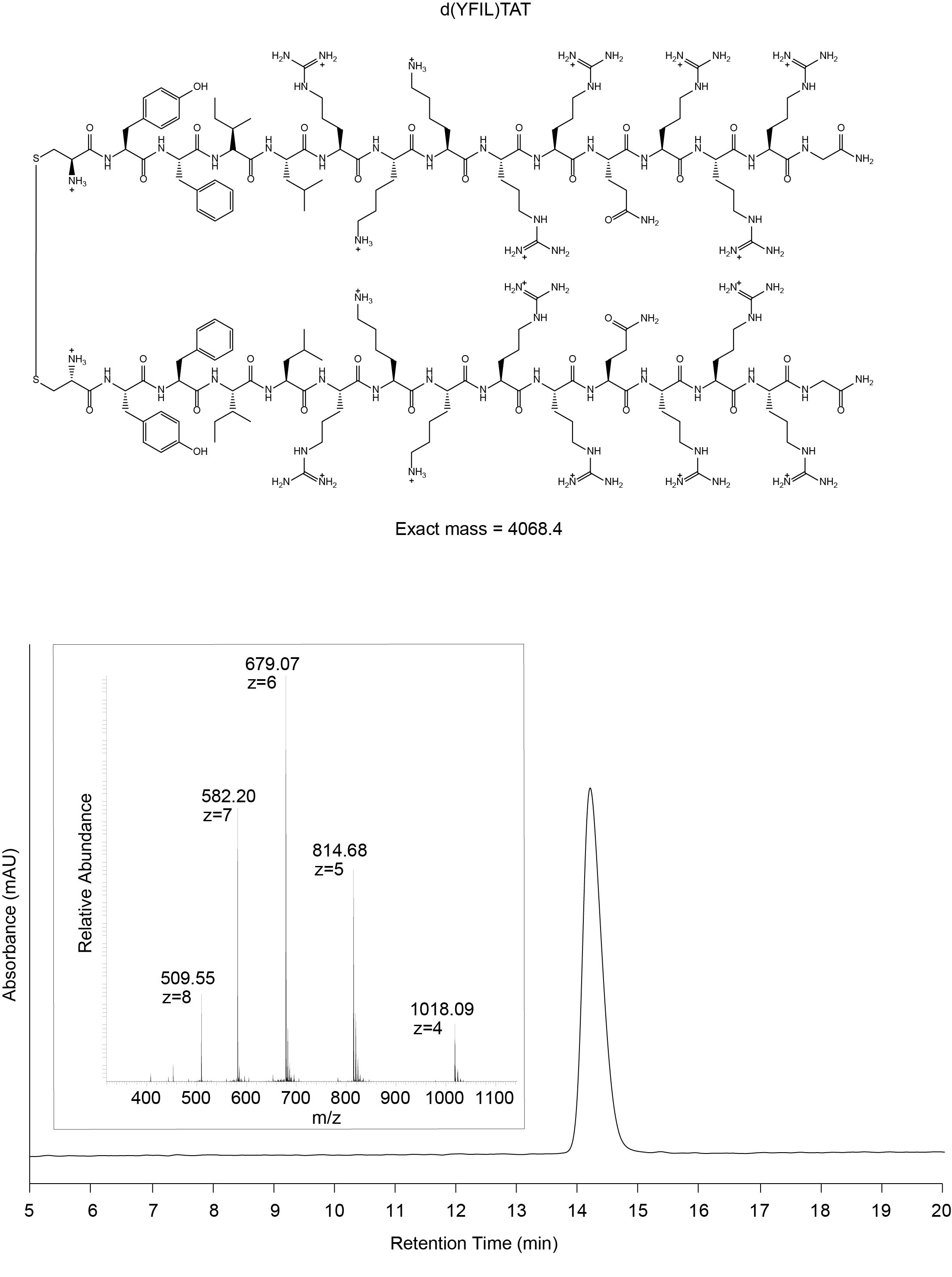


Figure S8.


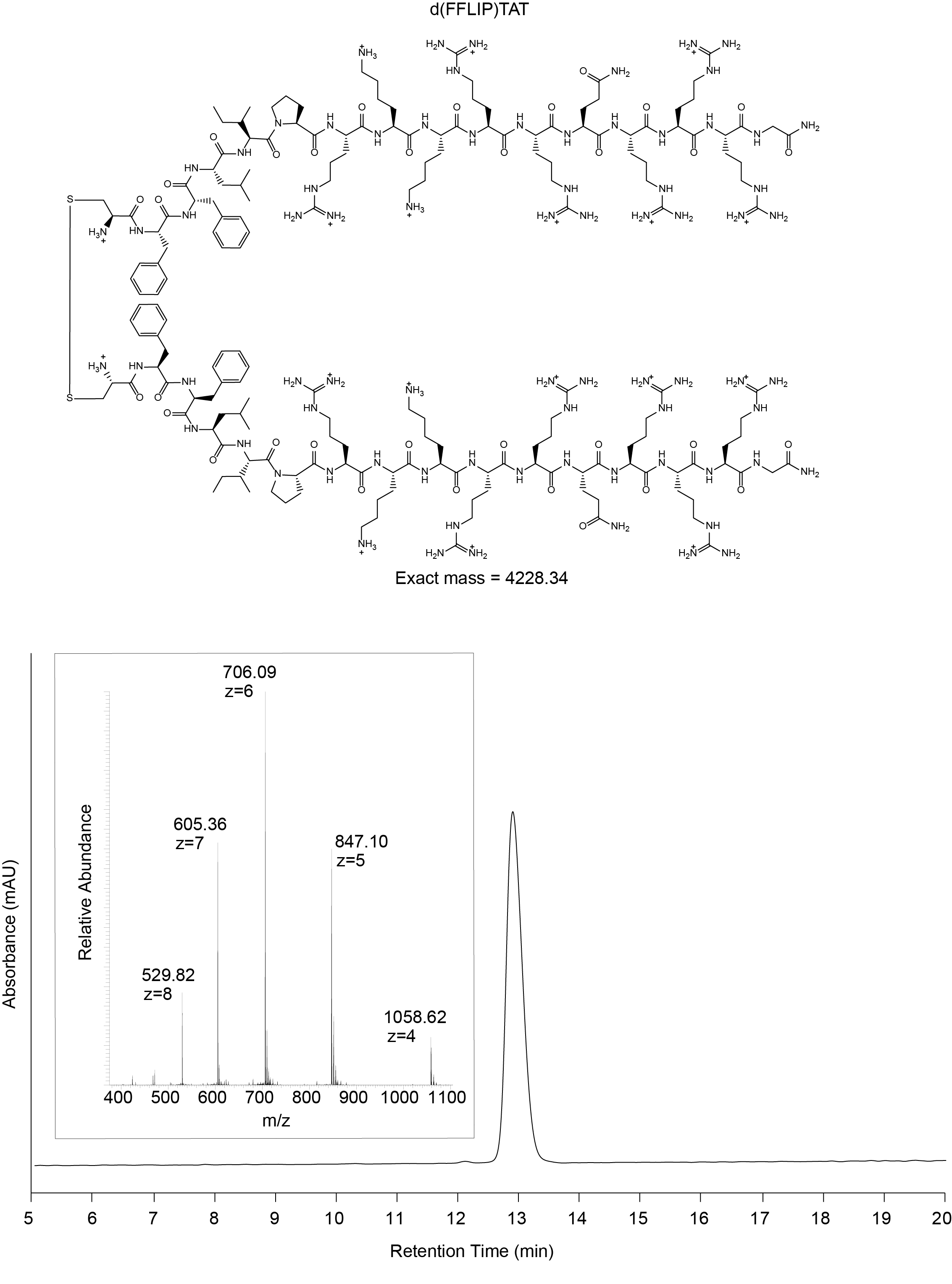


Figure S9.

Figure S10.

Figure S11. Chemical structures of the Ac-(X)-NH2 molecules synthesized and characterized. The M+H^+^ mass obtained by electrospray ionization on a Thermo Exactive Orbitrap is provided, along with calculated exact mass.


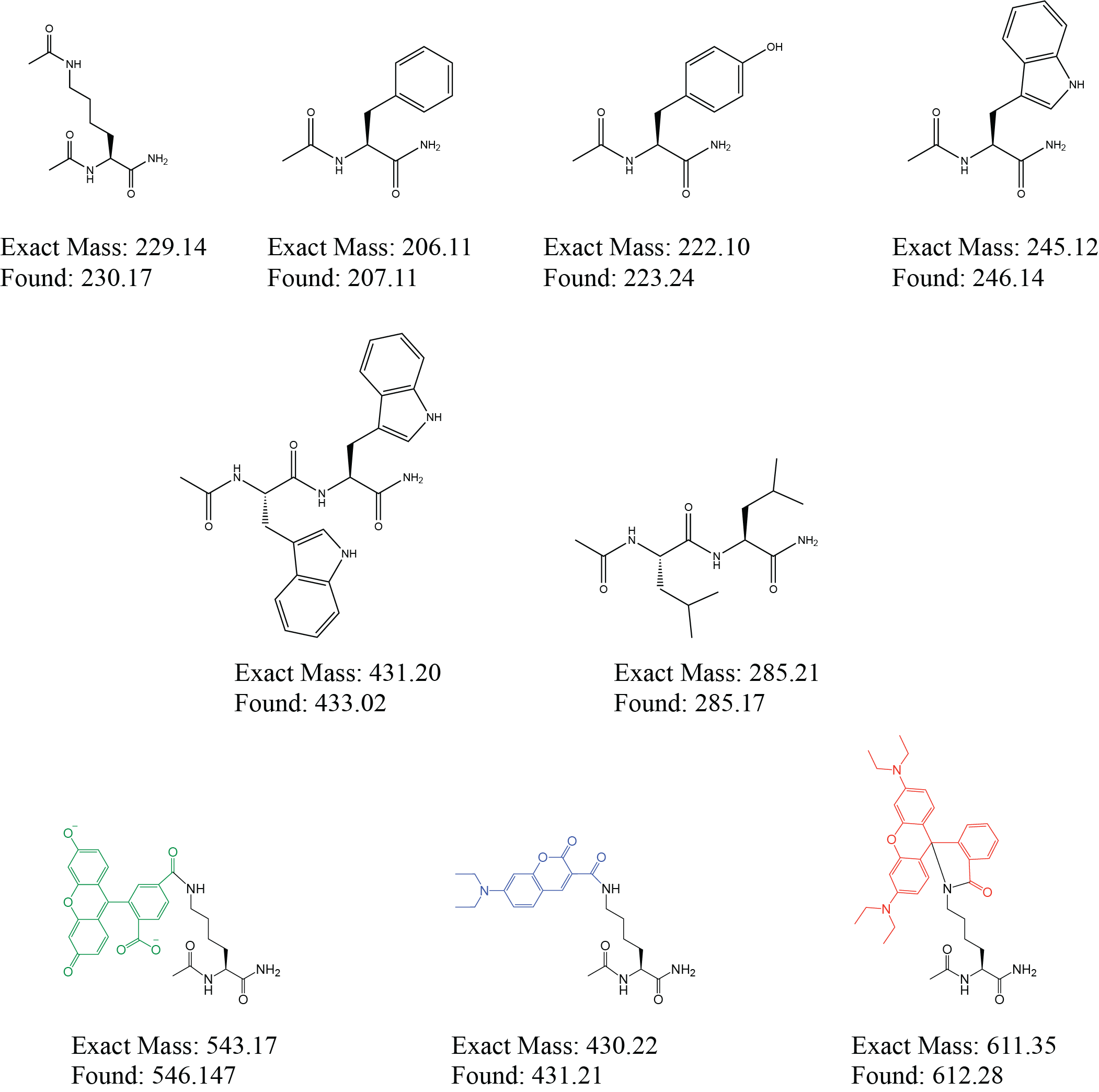


Figure S12. Neuro-2a cells were incubated with TMR-k5 (20 μM, 1 h), washed, and stained with Hoechst and LysoTracker green. Lysotracker green is a marker of acidic organelles, i.e. late endosomes and lysosomes. The Manders’ overlap coefficient R (measures how interdependent the red and green channels are) and colocalization coefficient M1 (measures the percentage of above-background pixels in the red channel that overlaps with above-background pixels in the green channel) were calculated using ImageJ (NIH). Please note that not all red puncta are green and vice versa. This is likely due to TMR-k5 being present in endosomes other than late end endosomes (e.g. endocytic vesicles and early endosomes that would not be stained by lysotracker). This is also in part due to endosomes moving between acquisition of the red and green images. Finally, please note that colocalization between green and red puncta increases with time, as previously reported (Brock *et al*., Cell Chem Bio, 2020). This is consistent with progressive accumulation of TMR-k5 into lysosomes as endosomal maturation progresses.


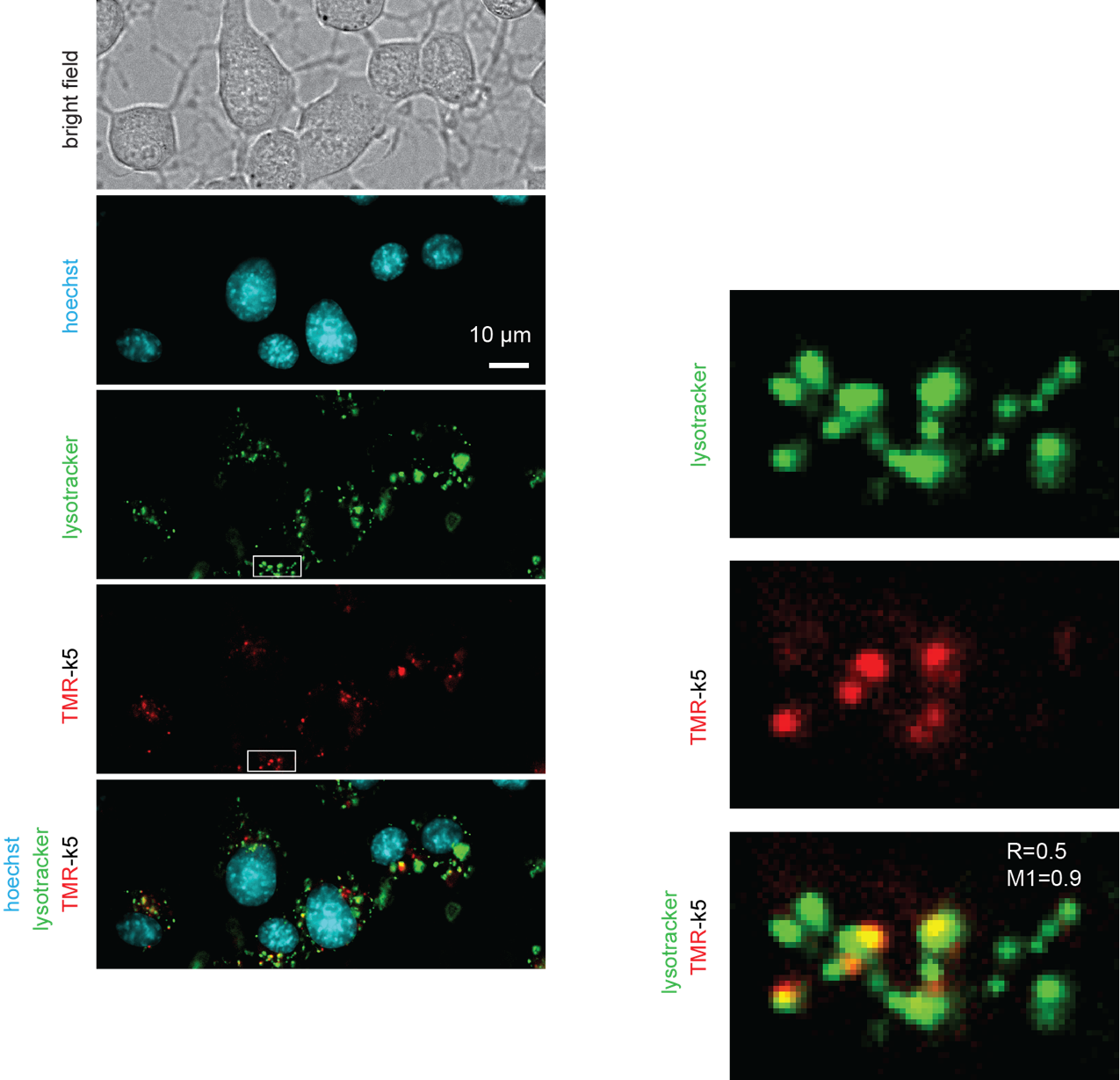


Figure S13.


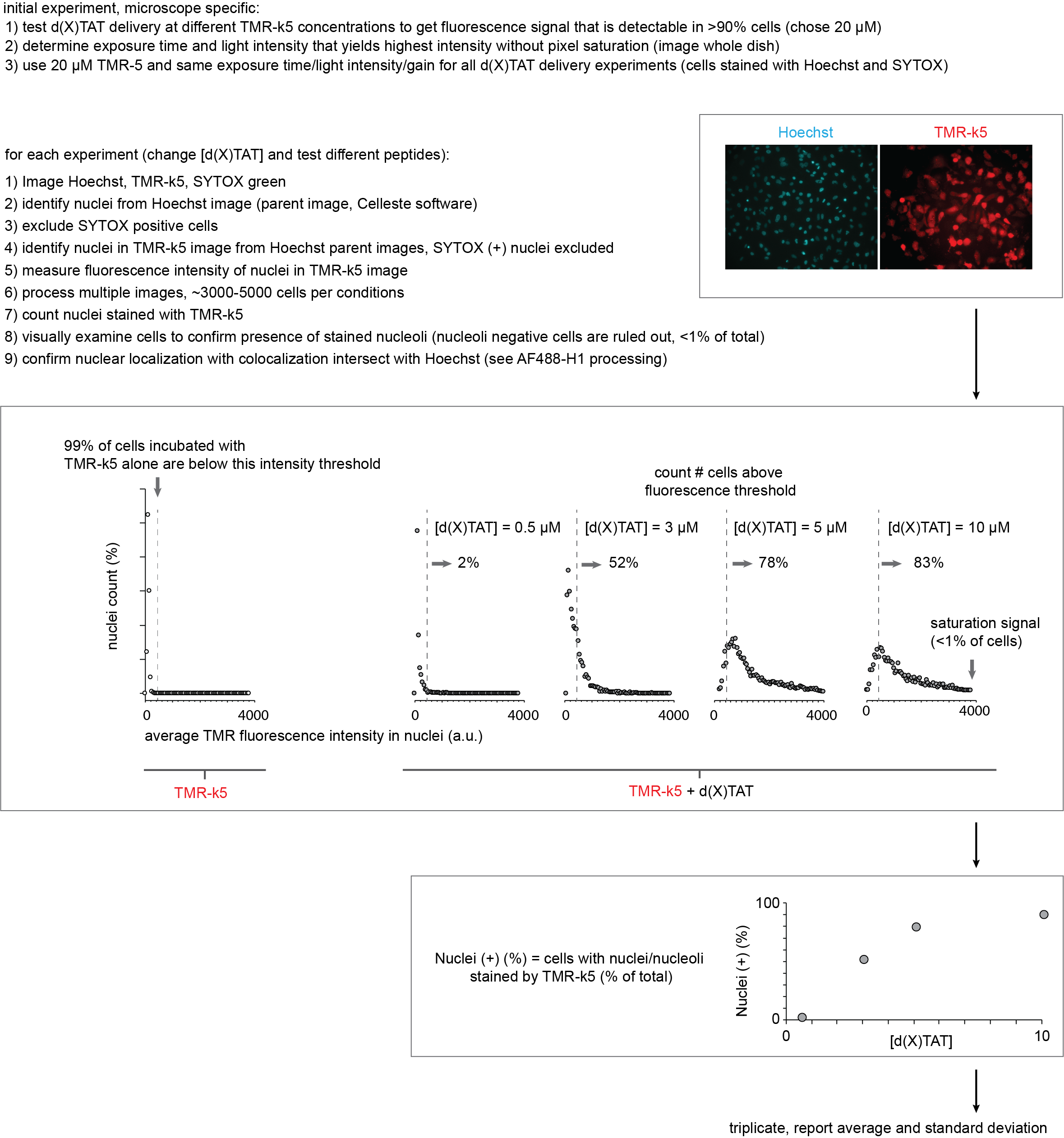


Figure S14. Comparison of the TMR-k5 delivery efficiency of d(X)TAT analogs in MDA-MB-231 and Neuro-2a. The corresponding toxicities, as established by counting the percentage of cells stained with SYTOX green, are also provided for both cell lines.


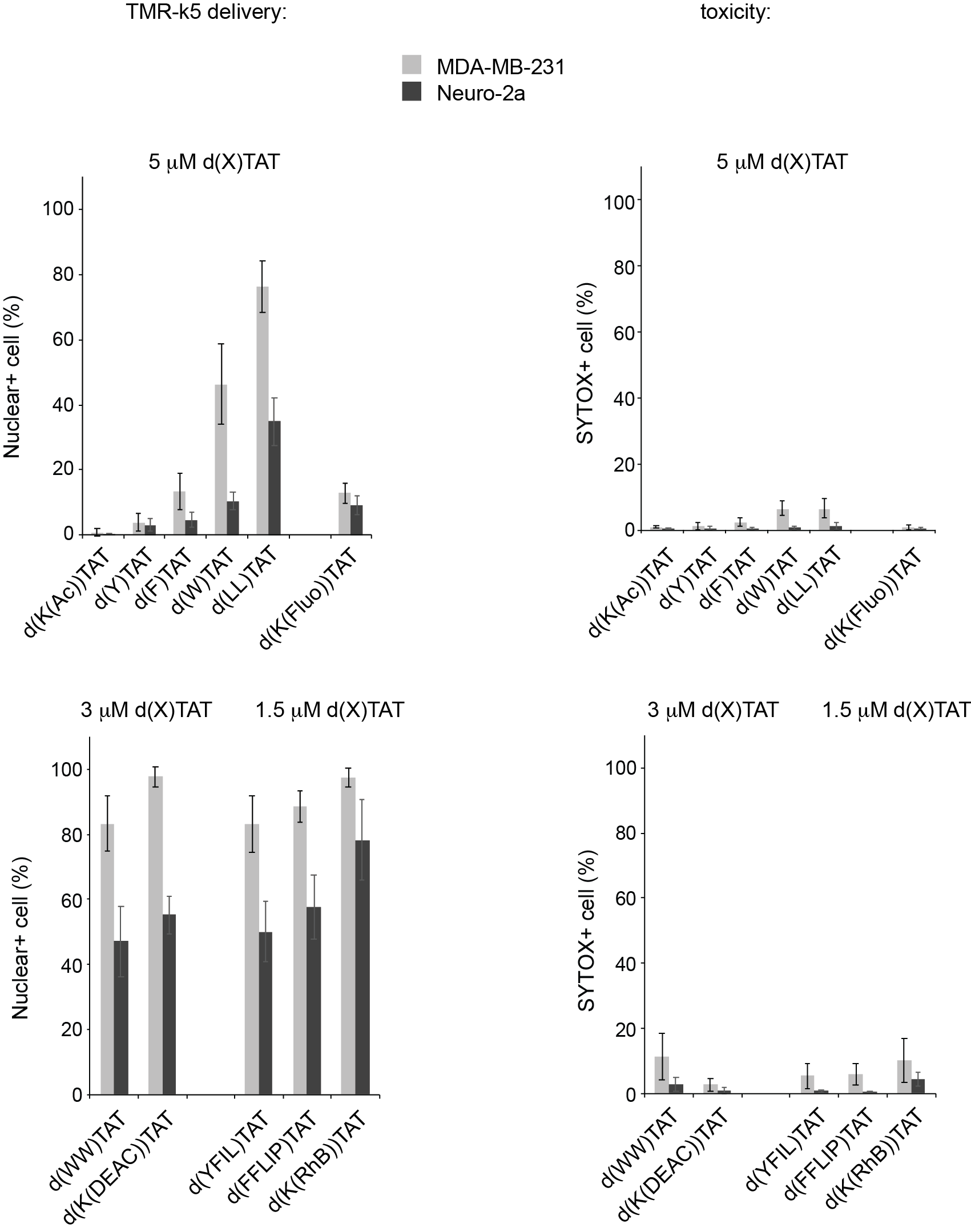

Supplement: Supplementary file 1 — Supplementary Information. [file 41598_2022_20425_MOESM1_ESM.docx]
